# Smart Floor Mats for a Health Monitoring System Based on Textile Pressure Sensing: Development and Usability Study

**DOI:** 10.2196/47325

**Published:** 2023-08-07

**Authors:** Hyunsoo Kim, Seong Jin Jang, Hee Dong Lee, Jae Hoon Ko, Jee Young Lim

**Affiliations:** 1 Department of Advanced Textile Research and Development Korea Institute of Industrial Technology Ansan Republic of Korea

**Keywords:** analysis, auto-mapping, monitoring, healthcare, health-monitoring, online, piezo-resistance sensor, pressure mat, real-time, sensing mats, smart home technology, smart home, spatial map, technology, textile

## Abstract

**Background:**

The rise in single-person households has resulted in social problems like loneliness and isolation, commonly known as “death by loneliness.” Various factors contribute to this increase, including a desire for independent living and communication challenges within families due to societal changes. Older individuals living alone are particularly susceptible to loneliness and isolation due to limited family communication and a lack of social activities. Addressing these issues is crucial, and proactive solutions are needed. It is important to explore diverse measures to tackle the challenges of single-person households and prevent deaths due to loneliness in our society.

**Objective:**

Non–face-to-face health care service systems have gained widespread interest owing to the rapid development of smart home technology. Particularly, a health monitoring system must be developed to manage patients’ health status and send alerts for dangerous situations based on their activity. Therefore, in this study, we present a novel health monitoring system based on the auto-mapping method, which uses real-time position sensing mats.

**Methods:**

The smart floor mats are operated as piezo-resistive devices, which are composed of a carbon nanotube–based conductive textile, electrodes, main processor circuit, and a mat. The developed smart floor system acquires real-time position information using a multiconnection method between the modules based on the auto-mapping algorithm, which automatically creates a spatial map. The auto-mapping algorithm allows the user to freely set various activity areas through floor mapping. Then, the monitoring system was evaluated in a room with an area of 41.3 m^2^, which is embedded with the manufactured floor mats and monitoring application.

**Results:**

This monitoring system automatically acquires information on the total number, location, and direction of the mats and creates a spatial map. The position sensing mats can be easily configured with a simple structure by using a carbon nanotube–based piezo-resistive textile. The mats detect the activity in real time and record location information since they are connected through auto-mapping technology.

**Conclusions:**

This system allows for the analysis of patients’ behavior patterns and the management of health care on the web by providing important basic information for activity patterns in the monitoring system. The proposed smart floor system can serve as the foundation for smart home applications in the future, which include health care, intelligent automation, and home security, owing to its advantages of low cost, large area, and high reliability.

## Introduction

Recently, the increase in single-person households has led to the emergence of social issues such as loneliness and isolation, commonly known as “death by loneliness.” The reasons for the increase in single-person households are diverse, including a growing desire for independent living and difficulties in communication between family members due to social changes. However, the increase in single-person households has also led to an increased risk of loneliness and social isolation, which can result in a death due to loneliness. In particular, older individuals who live alone are more likely to experience loneliness and social isolation due to difficulties in communication within the family and a lack of opportunities for social activities. Therefore, addressing the issues of single-person households and deaths due to loneliness is an important social issue that requires proactive solutions in our society. It is necessary to explore various measures and solutions to address these issues.

Nowadays, information regarding human behavior has gained increased interest owing to the development of internet of things (IoT) technologies and wireless network systems [1,2]. For example, virtual reality devices and intelligent monitoring cameras can be used to wirelessly detect and recognize human movement while playing games or monitoring movement [3-6]. Particularly, a new health monitoring system must be developed for the nursing field to obtain information on dangerous situations based on the movements of patients [7-11]. However, the existing monitoring devices restrict movement since they require tracking devices to be worn on the body or always carried. Therefore, a smart floor monitoring system, which obtains sensory information from human activities such as walking, position detection, and activity status, has gained considerable attention for the home training and health monitoring of patients at home or in hospitals without wearing or carrying devices [12,13].

The smart floor sensors adopted in the monitoring systems typically involve piezo-resistive, piezo-capacitive, and piezo-electric or triboelectric mechanisms [14-22]. When a person walks on a mat that is embedded with piezo sensors, the electric changes that are induced by the stepping action can be acquired and then used to obtain the person’s position. The existing smart floor systems employ a pressure sensing method that detects sitting or standing posture and movement by analyzing sensing data such as pressure, distribution, and shape [23-31]. However, these systems could not achieve large-area detection despite implementing highly sensitive floor sensors [23,26,32,33]; furthermore, the fabrication method used in these systems was complicated.

In this study, we have developed an affordable textile based on large-area smart floor mats (SFM), which uses a simple fabrication method, and established a system that monitors real-time behavioral information. The SFMs are operated as piezo-resistive devices, which are composed of a conductive textile, electrodes, main processor circuit, and a mat. The developed smart floor system acquires real-time position information using a multiconnection method between the modules based on the auto-mapping algorithm, which automatically creates a spatial map. The auto-mapping algorithm allows the user to freely set various activity areas through floor mapping, such as the living area, sleeping area, toilet area, and entrance. Furthermore, the developed smart floor mats present high durability and stability, which are demonstrated by 50,000 repeated presses. Lastly, the performance of the proposed smart floor system is evaluated by detecting various behavioral patterns of the subject. The system is evaluated in a room with an area of 41.3 m^2^, which is embedded with the manufactured floor mats. It is observed that the system presents high accuracy. Therefore, the behavioral information analysis data of the SFM can be applied to the cloud system, the information on the health and safety of the patients or single-person households can be predicted, and a system with an alert function can be configured.

## Methods

### Fabrication of the Piezo-Resistive Textile

The piezo-resistive textile (Duek Keum, Korea) is fabricated by using 100% polyester 400D, which is coated with a multiwalled carbon nanotube (MWCNT) through the knife coating method. The MWCNT coating solution is composed of 1.5% MWCNT and 4% polycarbonate-based polyurethane, with a viscosity of 15,000 cps. The knife coating was performed twice, maintaining a distance of 1 mm between the cloth and the knife, and dried at 150 °C.

### Electric Characterization

The change in the resistance of the piezo-resistive textile was measured using a multimeter (U1282A, Keysight) and oscilloscope (DSOX4024A, Keysight). The hysteretic behavior during the loading and unloading cycles was measured at a speed of 1 mm/minute from 0 to 0.08 kg/cm^2^ using a universal testing machine (Instron). The stability of the piezo-resistive sensor was measured by stepping on the mat 50,000 times with a weight of 55 kg.

### Textile Based–Pressure Sensing Mat System

The textile based–pressure sensing mat is assembled as a layered structure using 2 urethane mats, 4 electrodes, a circuit, and piezo-resistive textile. The custom-made urethane mats (500×500 mm) were fabricated by using a mold system (Poled). The electrode plate (220×220 mm) is coated by a lead/tin (Pb/Sn) alloy in an alternating pattern of positive and negative electrodes on the phenol plate. The main processor uses the ATMEGA32M1 IC from Microchip Technology. The ATA6561-GAQWIC from Microchip Technology is used for control area network (CAN) communication. The circuit is manufactured to enable 4-point pressure measurement since there are 4 pressure measurement zones on 1 mat. Therefore, it is designed to connect the 4 electrode plates and the 4 connecting wires to the circuit. A total of 57 assembled SFMs were connected to each other, and a gateway was connected to the first mat. The voltage used for the power source is 6 V. The real-time behavior information is programmed using Unity, which is an integrated production tool used to create web-based content.

### Ethics Approval

This research is approved by the institutional review board (IRB) of the Korea National Institute for Bioethics Policy (P01-202205-01-003). To protect the personal information of the participants, we did not record any details such as names, ages, or addresses. During the pressure mat experiment, we covered the faces with masks to ensure that they were not exposed in the recordings. The camera used for the recordings strictly prohibited the use of personal devices, and only designated cameras were allowed. All experimental data was stored on a computer at the Korea Institute of Industrial Technology (KITECH), which was secured with a password.

## Results

### Potential Application of the Smart Floor System

**Figure 1:**
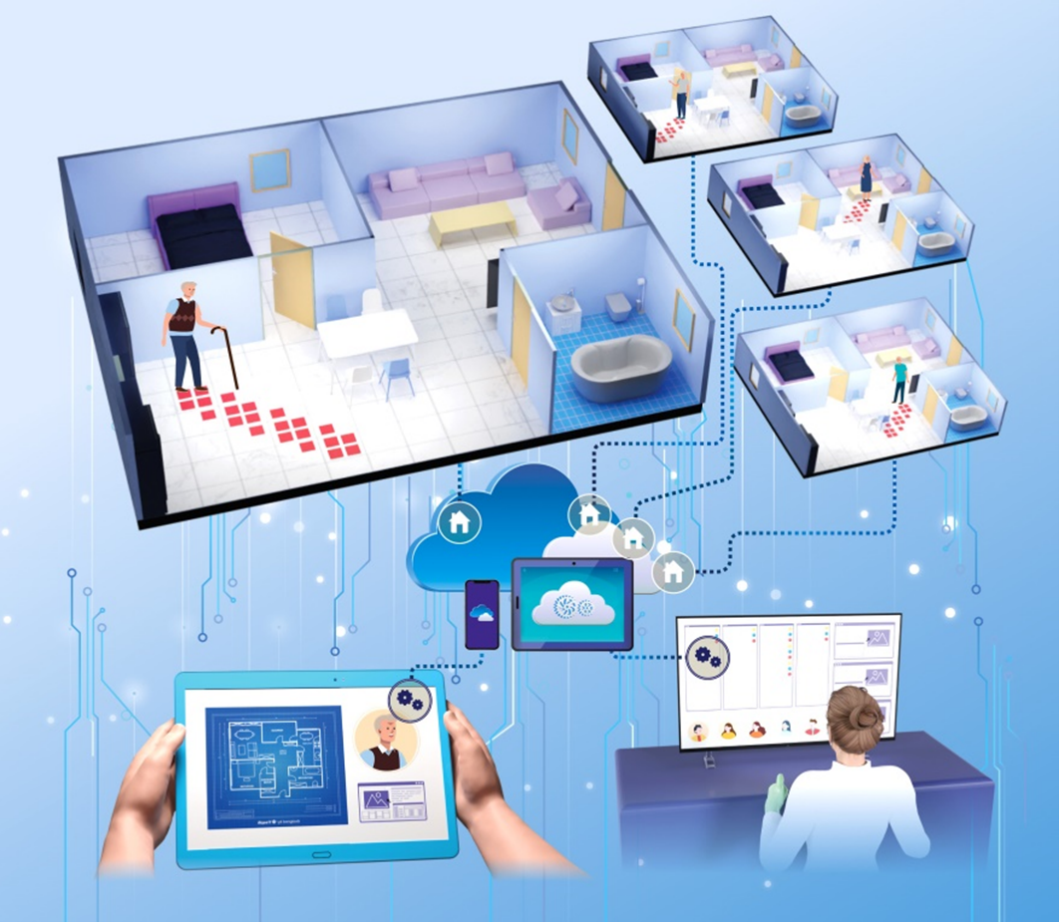
Schematic diagram depicting a potential application of the smart floor system. Behavioral patterns can be analyzed using position information to monitor health status. Additionally, behavioral information can be managed on the web to effectively manage health.

Figure 1 depicts a potential application of the SFM for the health care monitoring system in a smart home. When a person walks into the smart home, real-time behavior information is obtained and transmitted to the monitoring system by SFMs, which communicate between the gateway and the piezo-resistive sensors. Therefore, the activity information of the patient to be observed is automatically transmitted to the caregiver. Therefore, an effective and personalized health management system can be created to monitor information such as the health status and provide exercise guidance through the transmitted position information. Additionally, patient behavior information can be remotely accessed, and alerts regarding abnormalities can be provided by managing the information on a cloud server. In the future, smart floor systems with position sensing can be developed to improve the quality of life by providing more effective home care services, including health care, exercise, security, and IoT.

### Smart Floor Mat

**Figure 2:**
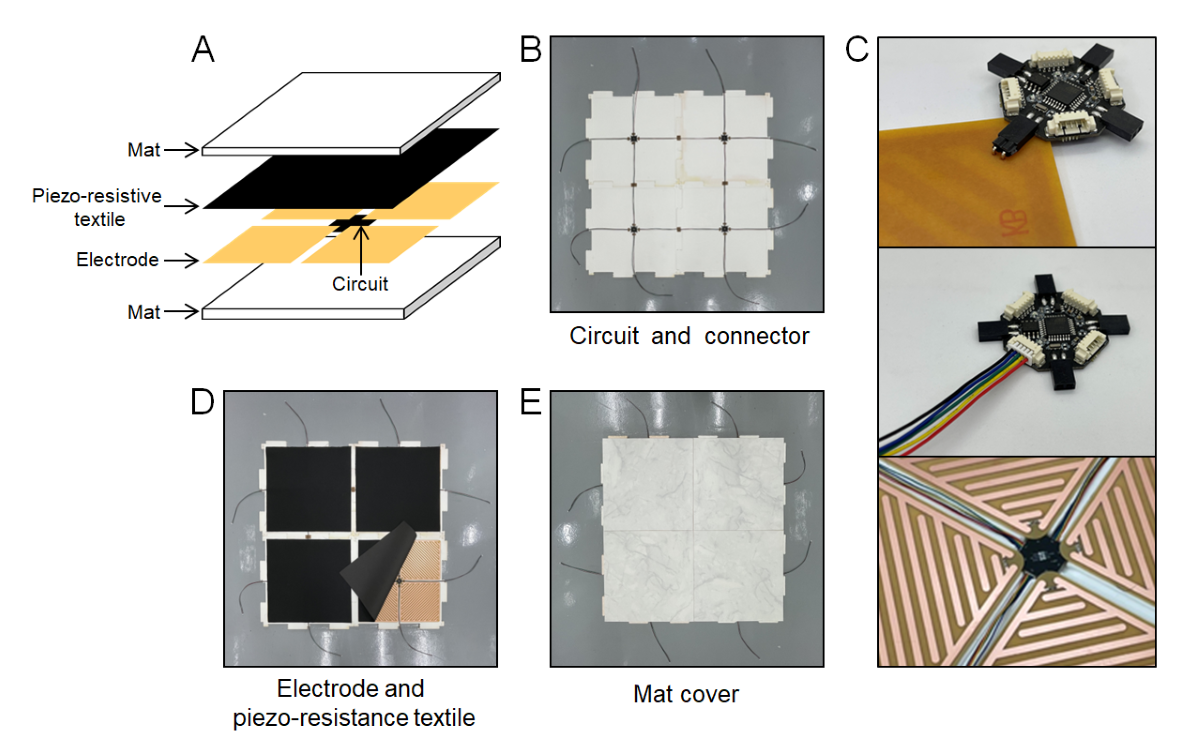
Structure of the smart floor mat (SFM). (A) Schematic diagram of the SFM layer structure. Photographs of (B) bottom mats+MCU (microcontroller unit) circuits, (C) connection between MCU circuit and electrode plates and 5-pin connector, (D) piezo-resistive textiles on the bottom mat+MCU circuit and electrode plates, and (E) upper mats piezo-resistive textiles+bottom mat + MCU circuit and electrode plates.

Figure 2 presents the SFM developed for the health care monitoring system. It consists of a 4-layer structure comprising a mat, a piezo-resistive textile, and a microcontroller and electrodes layers (Figure 2A). A microcontroller unit (MCU), which can transmit a digital signal of the resistance change, was installed in the center of the mat to communicate with the gateway (Figure 2B). The MCU is designed to connect the 4 electrode plates and the four 5-pin connecting wires (Figure 2C). The 5-pin connector includes 2 wires for the power supply, 2 wires for CAN communication, and 1 wire for auto-mapping to establish a connection between the MCUs. In the case of the electrode plates, the Pb/Sn alloy is printed on the phenol plate in an alternating pattern of positive and negative electrodes (Figure S1 in Multimedia Appendix 1). The 5-pin connecting wire and the Pb/Sn alloy-printed phenol electrode plate (210×210 mm) was connected to the MCU on the bottom urethane mat (500×500 mm). The electrode was then covered over the piezo-resistive textile and upper mat (Figures 2D and 2E). The SFMs uses a fire-resistant polyester material for the piezo-resistive textile. Additionally, the main circuit, directly connected to the power supply, is physically separated from the mats and operates at a low voltage of approximately 6V, thereby minimizing the risk of fire hazard.

### Pressure Sensing

**Figure 3:**
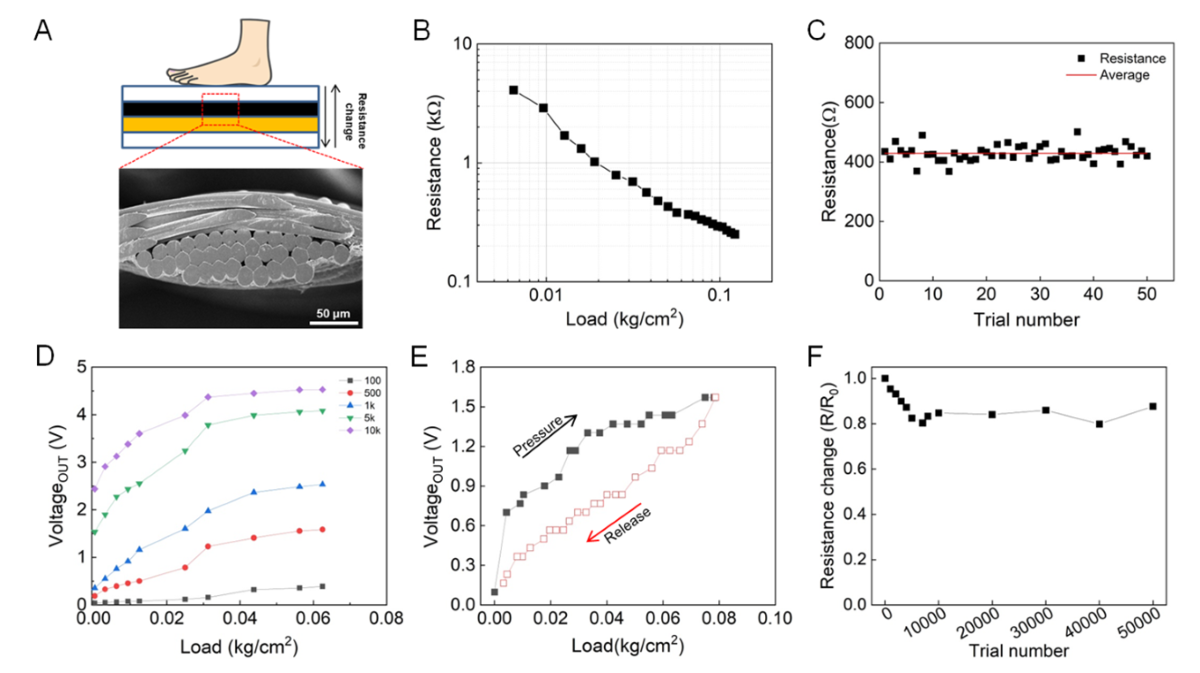
Electric characterization for piezo-resistive textile. (A) Cross-sectional images of the smart floor mat (SFM) and scanning electron microscope image of the piezo-resistive textile, which is coated with multiwalled carbon nanotubes (MWCNTs). (B) Resistance change based on load. The load is increased from 0 to 0.12 kg/cm2. (C) The change in resistance is measured repeatedly at a specific weight (0.05 kg/cm2). (D) Force versus voltageout curves based on load resistance (a voltage of +5V was used). (E) Hysteresis curve of the piezo-resistive textile. (F) Durability for repeated pressure 50,000 times.

The change in pressure was measured based on the piezo-resistive effect of the textile, which is coated with MWCNT, as shown in Figure 3A. The MWCNTs are coated on 1 side of the textile with a thickness of ~230 μm (Figure S2 in Multimedia Appendix 1). When pressure is applied on the mat, the conductivity increases with the increase in the density of MWCNTs, and the resistance decreases. Figure 3B depicts the change in resistance of the piezo-resistive textile based on weight. The resistance decreased from 4.1 kΩ to 0.25 kΩ with the increase in weight from 0 kg/cm^2^ to 0.12 kg/cm^2^. The accuracy of the piezo-resistive sensor was 92%, with an error of ±10% of the average resistance value at one specific weight (Figure 3C). Figure 3D depicts a family of force versus voltage curves for the resistive sensor in a voltage divider configuration with various resistors to determine the optimal load resistance value. The force versus voltage curves demonstrate that a 1 kΩ resistor presents the highest rate of change in voltage for the sensing force range of the piezo-resistive textile.

Figure 3E depicts the voltage versus pressure curve that showed hysteretic behavior during the loading and unloading cycles. Hysteresis is an important characteristic of the piezo-resistive materials for accurately measuring the signal. The degree of hysteresis (DH) can be defined as the difference in the area of the loading and unloading curves, and is calculated as follows:



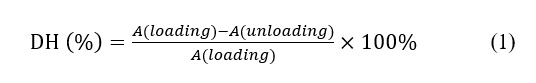



where A_(loading)_ and A_(unloading)_ represent the area of loading and unloading curves, respectively [34,35]. Our piezo-resistive sensor presented a DH value of 32.2% when the applied pressure changes from 0 kg/cm^2^ to 0.08 kg/cm^2^.

Piezo-resistive sensors typically exhibit a large hysteresis under the loading and unloading of pressure owing to the weak interaction between the conductive material and the substrate [34,35]. Figure 3F depicts the change in resistance after 50,000 presses to verify the stability of the resistance sensor. Initially, the resistance gradually decreased by 20%; it was then stabilized without any significant change in the resistance after the senor was pressed approximately 8000 times. This characteristic can be attributed to the carbon nanotubes, which are gradually rearranged under pressure and deformed into a stable internal structure.

### Auto-Mapping Algorithm

**Figure 4:**
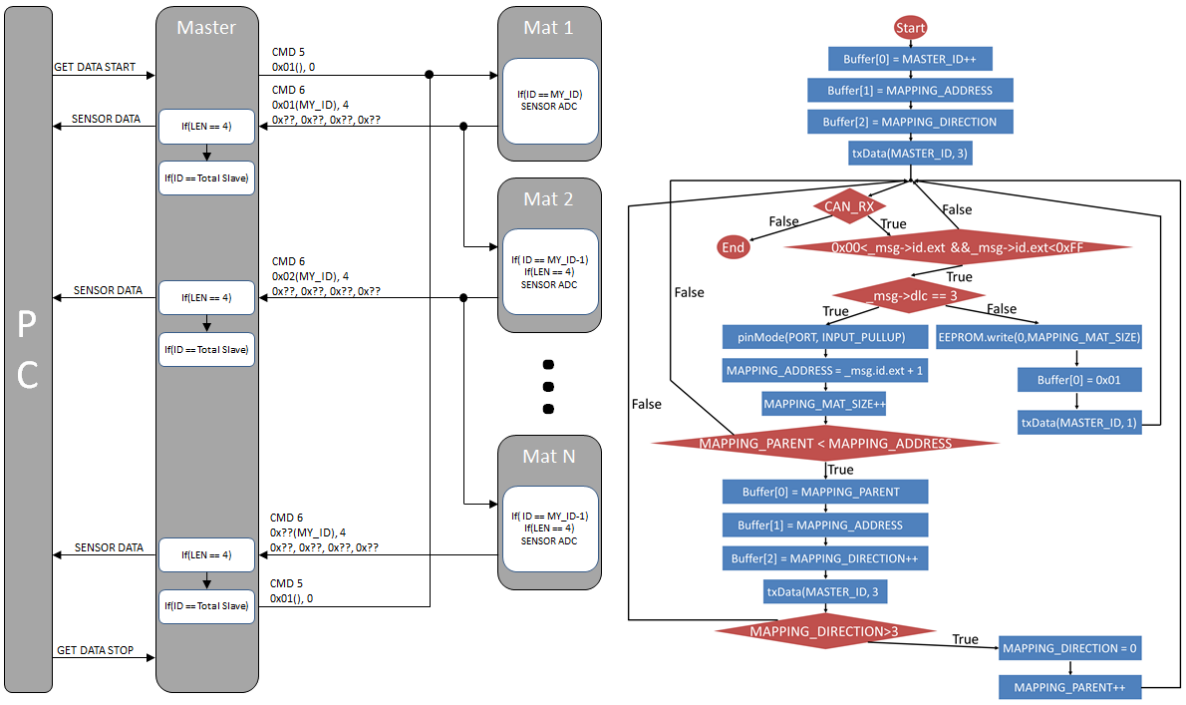
Schematic of diagram (left) and algorithm (right) for control area network (CAN) communication and algorithm of auto-mapping process.

We implemented the CAN communication method to construct the smart floor system to ensure that the microcontrollers or devices could communicate with each other without a host computer (Figure 4). This method can be used to randomly connect multiple SFMs and process data since it is a multimaster network; additionally, it is inexpensive and easy to procure. It must manually set the address for each mat to communicate between the gateway and SFMs due to the direction and position change variables while installing the mats. Therefore, we have developed an auto-mapping method in which the gateway automatically identifies the total number, location, and direction information of the SFMs and creates a spatial map. The MCU of the SFMs contains ports for auto mapping in 4 directions (east, west, south, and north). The port waits in an input state during auto mapping, and when a low signal is received, the number of the port that received the low signal is transmitted to the gateway. Subsequently, the gateway analyzes the data received from the MCU, addresses the MCU, and draws a map. The auto-mapping port of the addressed MCU is set to output, and the signal of the port is sequentially converted to low to detect the surrounding MCU (Figure 4). Due to this auto-mapping process, each column and row mat number is specified as follows: 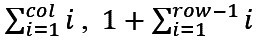 (Figure S3 in Multimedia Appendix 1).

## Discussion

### Position Sensing

**Figure 5:**
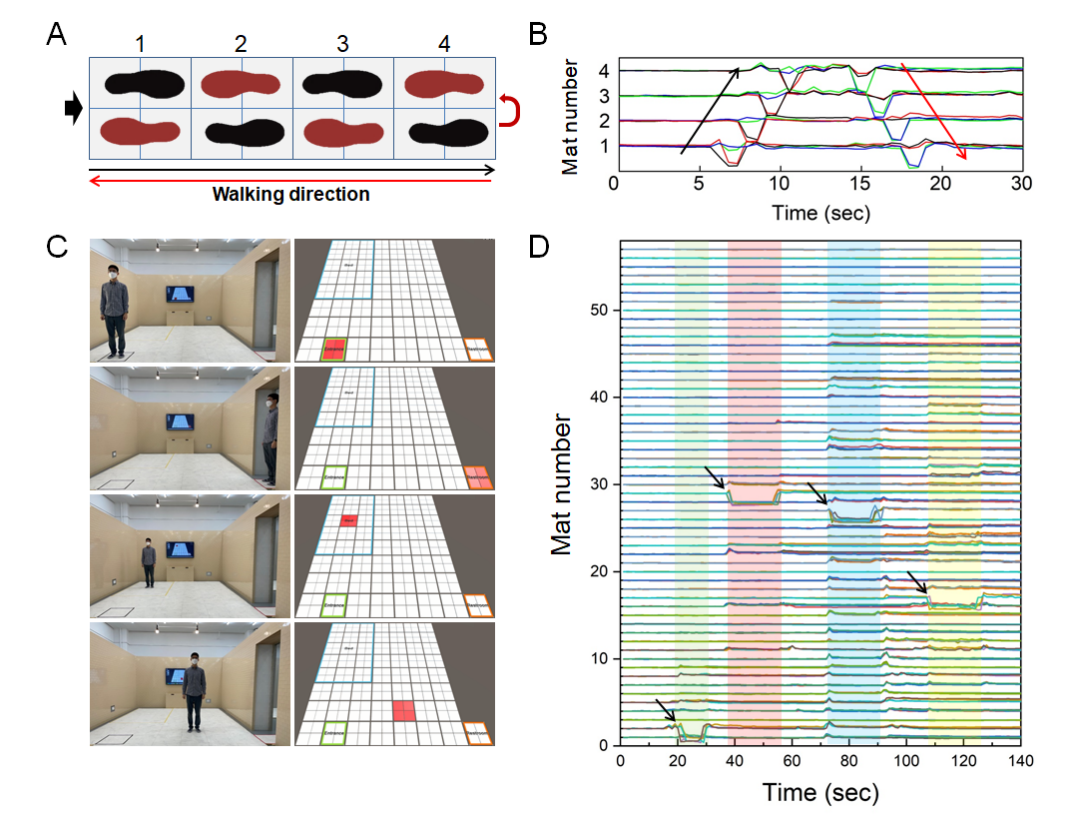
Analysis of the assembled smart floor mats (SFMs). (A) Schematic diagram of the parallel connection with a 1×4 arrangement. (B) The generated output signals by forward-backward walking on the 1×4 arrangement SFMs. (C) Pictures of the person standing in each area (bedroom, toilet, entrance, and other activity area) and the captured images showing the location of the person (depicted in red). (D) The generated output signals of the 57 SFMs when standing in each area (Green: entrance, red: toilet, blue: bedroom, yellow: activity area).

Figure 5 depicts the output performance of the SFM to obtain walking and position sensing information. A single SFM contains 4 electrode plates (sensing area); therefore, the area is divided into 4 equal parts and 4 output signals are generated. The smaller the electrode plates and the greater the number of electrode plates, the more detailed the location information. However, an increase in the number of construction procedures reduces the sensors’ reaction time. The 4 electrode plates that could reduce the effect on the response speed and location information were determined to obtain accurate and real-time location information. Figures 5A and 5B depict the output signals, which are converted from the resistance value to digital signals. They represent a person performing a simple walking action on the mat array in a forward-backward manner. The walking activity can be easily distinguished by the change in the magnitude of the output signal over time. A large change in signal magnitude was detected only in the area stepped on by the person while walking in the forward-backward path, as shown in Figure 5B. Therefore, behavioral information such as a person’s location and time of stay can be determined based on the number of the SFM and the change in the signal.

Furthermore, the 57 SFMs were installed in a large area (a room with an area of 41.3 m^2^). We divided the house into bedrooms, toilets, entrances, and other activity areas to analyze the behavior information. We then created a program that can visually determine the location information of a person in the room based on the signal changes in the SFM. Figure S4 in Multimedia Appendix 1 presents the SFM numbers for the test room. Figure 5C presents the captured images of the person standing in each area, which is depicted in red. Similarly, the location and movement information can be obtained in real-time by matching the SFM number and signal (Figure 5D). Therefore, this program can be implemented to determine human movement and location information in real-time.

### Behavior Pattern Monitoring System

**Figure 6:**
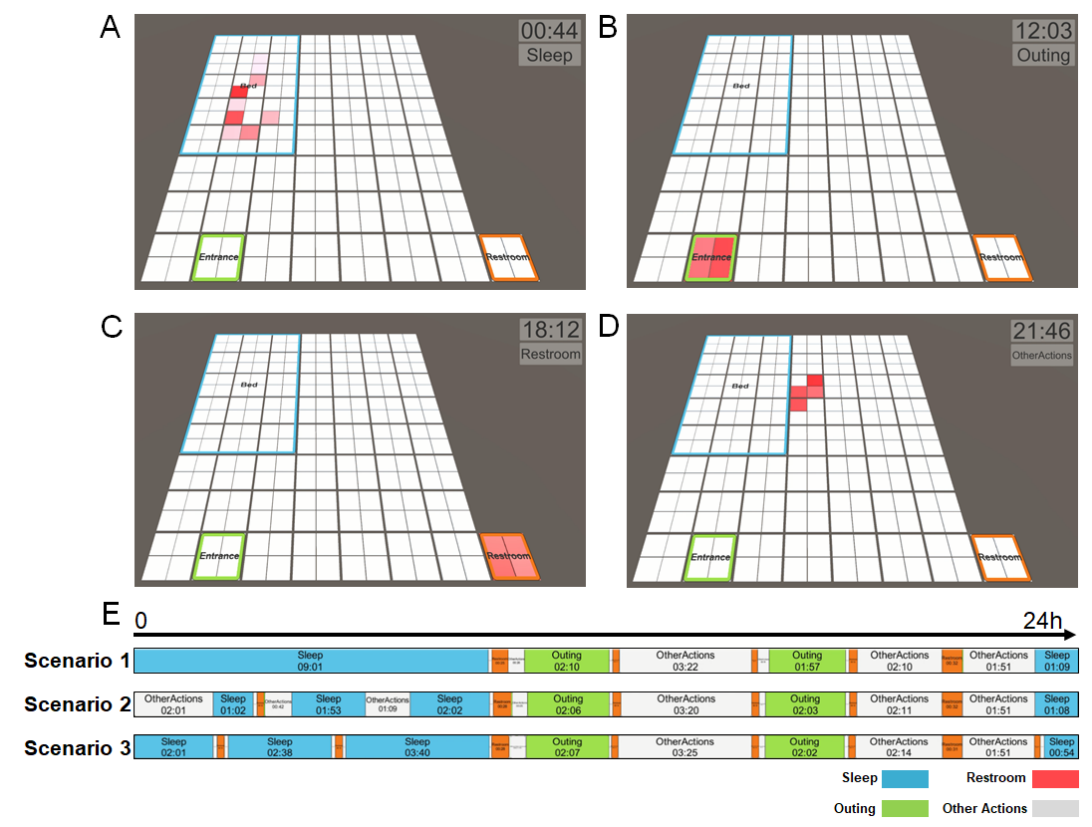
Behavior pattern monitoring system through scenarios. Images when standing in (A) bedroom, (B) entrance, (C) toilet, and (D) other activity area in a normal scenario. (E) Behavior patterns for 24 hours assuming normal (scenario 1), sleep disorder (scenario 2), and urinary frequency scenarios (scenario 3). The scenarios were performed for 24 minutes (24 hours) assuming 1 second as 1 minute.

Subsequently, we have added a function to view the activity information in real-time in the program and performed 3 scenarios: normal, sleep disorder, and urinary frequency. The sleep disorder and urinary frequency scenarios were performed based on the normal scenario, which was written arbitrarily. In the sleep disorder scenario, it was assumed that the person woke up 3 times during sleep. Since the person in the case of the urinary frequency typically uses the bathroom more than 8 times a day, the scenario was performed of the person going to the bathroom 8 times, including 2 times during sleep. Figure 6 presents the real-time behavioral information recorded for the 3 scenarios. We performed the scenario assuming 24 hours as 24 minutes and 1 second as 1 minute. Figures 6A-6D depict the images of the real-time position of the person in each zone in the normal scenario (the real picture is presented in Figure S5 in Multimedia Appendix 1). Compared to the normal scenario, it is observed that the person in the sleep disorder scenario woke up 3 times during sleep. Similarly, in the case of urinary frequency, 8 trips to the bathroom were shown in 24 hours including the 2 trips to the restroom during sleep. Therefore, the activity information (sleep, activity, and restroom time) obtained in real time using the SFM, and the program can be used to monitor the patient’s health. The behavior pattern matching accuracy between the real behaviors (the number of actions entered in each area) and the pattern recognized in the program was 100%, as shown in Table S1 in Multimedia Appendix 2. Consequently, we were able to accurately determine the behavioral patterns with a simple smart mat.

### Limitations

While the current system monitors behavior patterns accurately, more detailed behavior analysis (such as sleep state, exercise state, etc) requires additional sensors. In the future, by integrating sensors with pulse oximeters and motion detectors, it will enable more precise monitoring and diagnostics. Furthermore, the development of algorithms for distinguishing multiple individuals and the encryption of personal data are required. Therefore, in the next phase of research, software development, including activity monitoring systems using various sensors and activity member identification algorithms and personal data encryption techniques, will be conducted in collaboration with experts from respective fields. Within an integrated system, the SFMs will provide important data that will serve as the basis for analyzing behavioral patterns in the future.

### Conclusions

In this study, we present a pressure sensing–mat system for noncontact health care monitoring. It is achieved by integrating the textile based–piezo resistive sensor and the system of communication between the mats and gateway. The textile based SFM, incorporating an automated mapping technique that detects the quantity, positioning, and orientation of mats to generate a spatial map, offers various advantages such as cost-effectiveness, extensive coverage capability, and easy installation. Furthermore, we establish a noncontact monitoring system that accurately tracks a person’s location in real time using the SFMs. As a result, through the execution of various scenarios, we demonstrate the potential to assess differences in behavioral patterns by recording location information and time in real time. Therefore, the developed smart floor system will serve as an effective foundation for noncontact smart home care services, including health monitoring, daily management, and exercise.

## Appendix

Multimedia Appendix 1 Supplementary figures.

Multimedia Appendix 2 Supplementary table.

## Abbreviations

CAN: control area network
DH: degree of hysteresis
IoT: internet of things
IRB: institutional review board
KITECH: Korea Institute of Industrial Technology
MCU: microcontroller unit
MWCNT: multiwalled carbon nanotube
SFM: smart floor mat

## Data Availability

The data sets generated and analyzed during this study are available from the corresponding author on reasonable request.
