# Supplementary material for: Smart Floor Mats for a Health Monitoring System Based on Textile Pressure Sensing: Development and Usability Study
Source: JMIR Form Res. 2023 Aug 7;7:e47325. doi: 10.2196/47325 (PMC10442732; doi:10.2196/47325)
Supplement: Multimedia Appendix 1 [file formative_v7i1e47325_app1.docx]

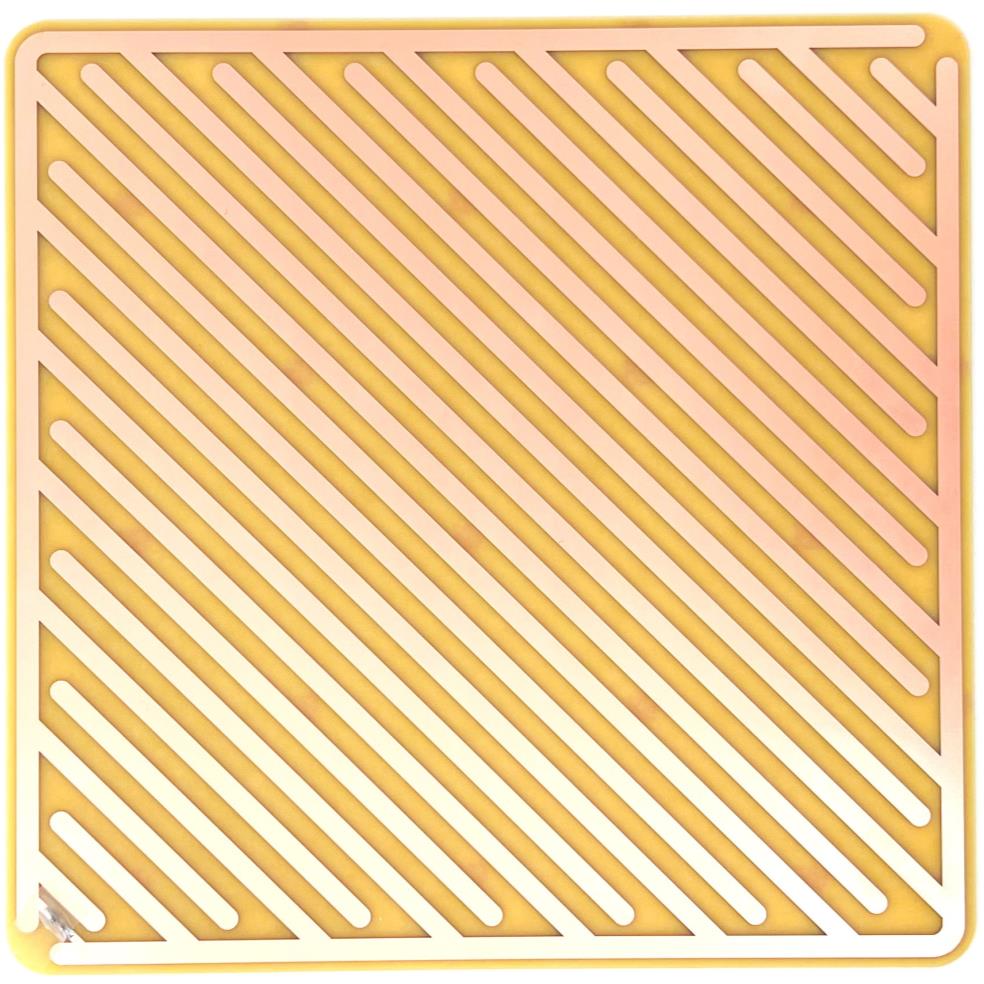


**Figure S1**. The Pb/Sn alloy coated phenol plate in an alternating pattern of positive and negative electrodes.


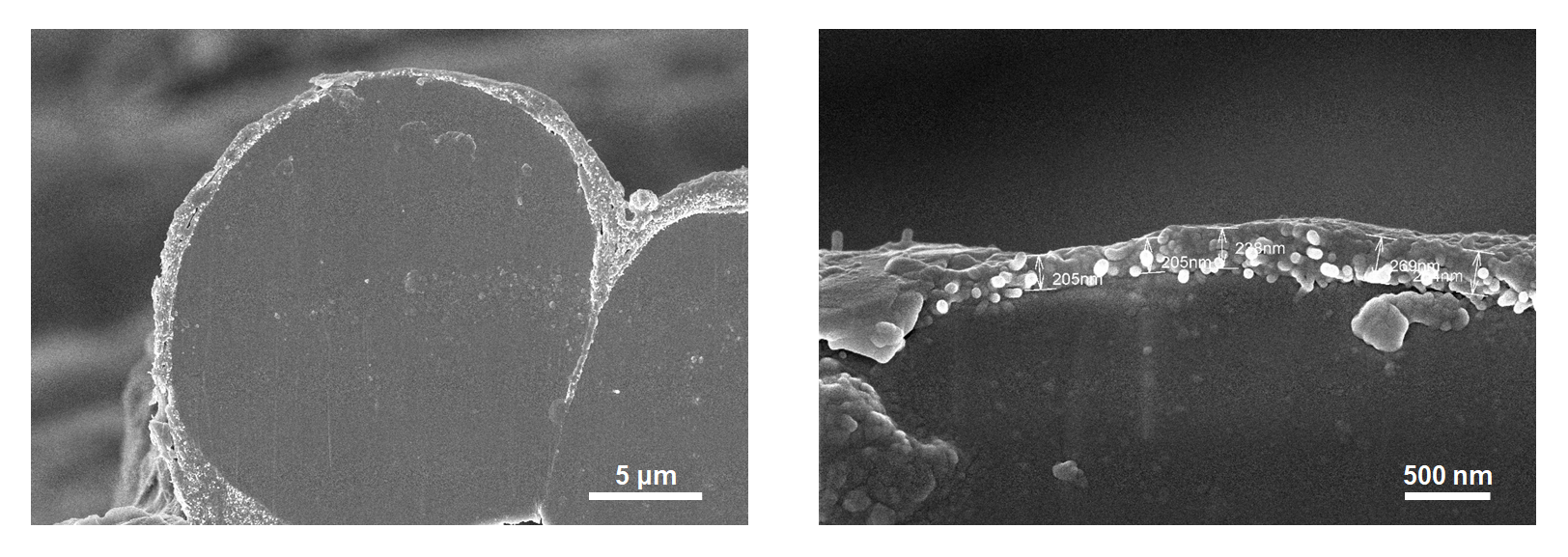


**Figure S2**. SEM images for cross-section of the MWCNTs coated textile.


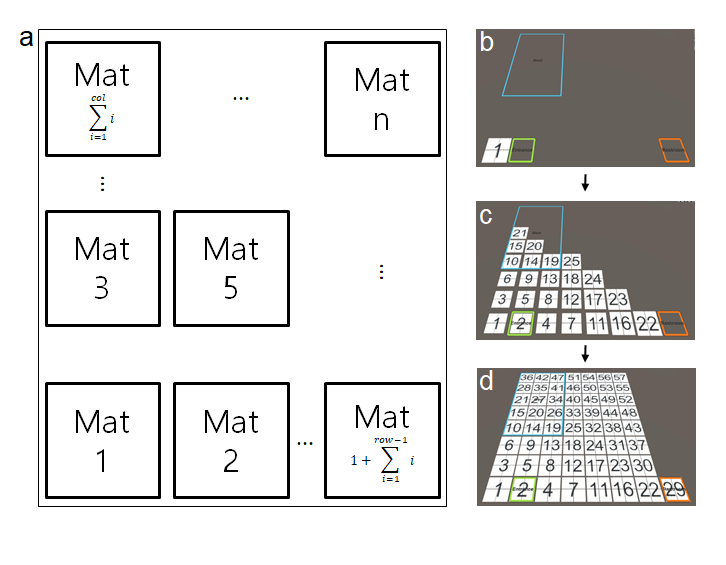


**Figure S3**. (A) Schematic diagram of the Controller Area Network system. (B-D) Images showing the auto-mapping process from number 1 to 57 of the SFMs to create a spatial map.


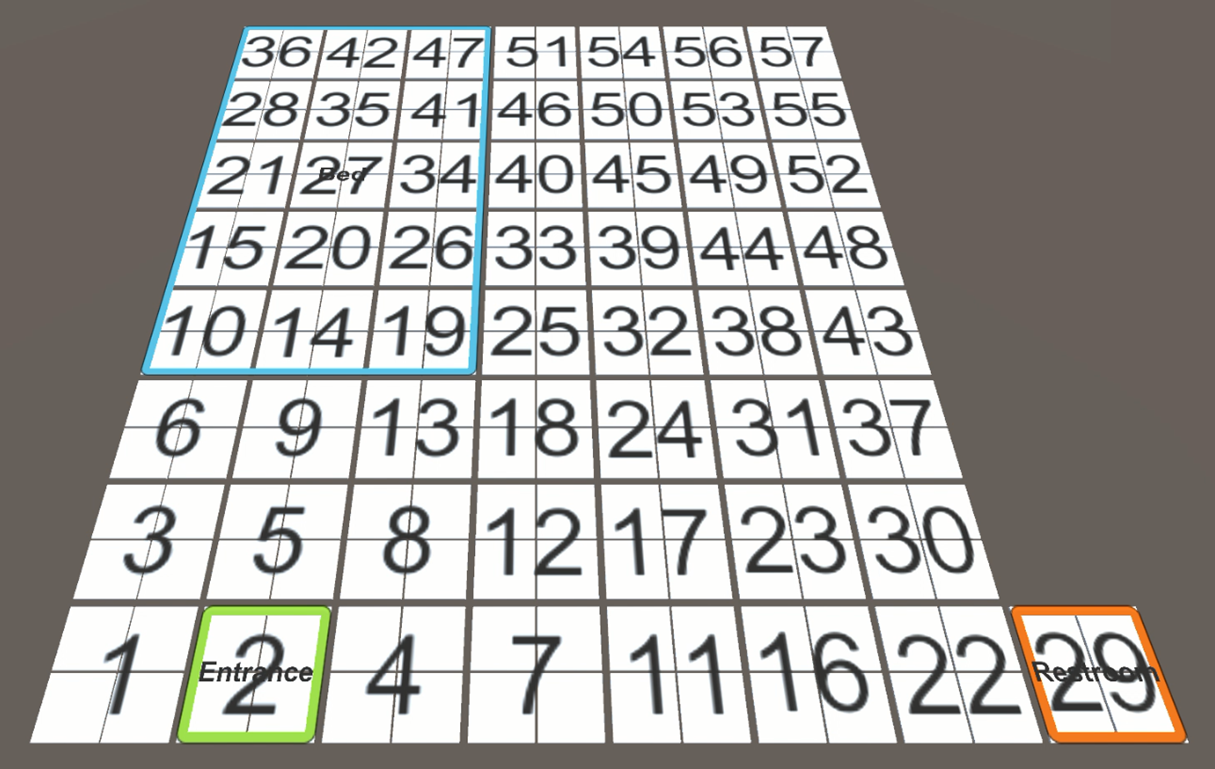


**Figure S4**. The SFM numbers for test room.


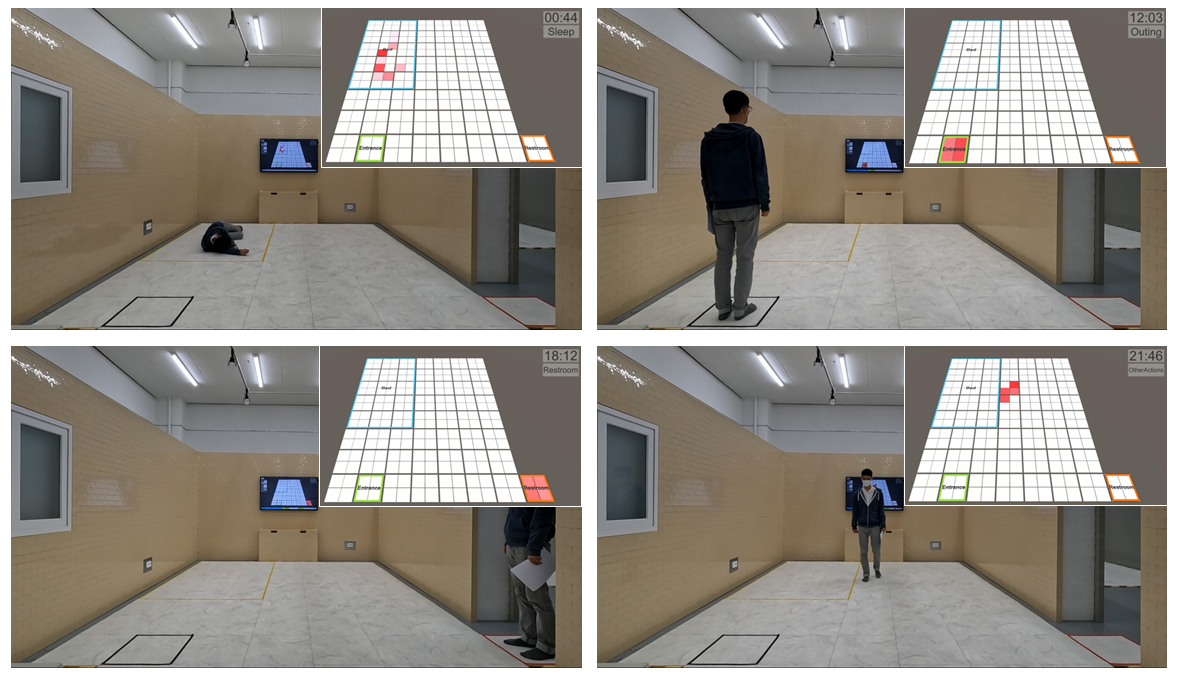


**Figure S5**. Photograph images for the real-time position when in each zone in the normal scenario.
