# Supplementary material for: Smart Floor Mats for a Health Monitoring System Based on Textile Pressure Sensing: Development and Usability Study
Source: JMIR Form Res. 2023 Aug 7;7:e47325. doi: 10.2196/47325 (PMC10442732; doi:10.2196/47325)
Supplement: Multimedia Appendix 2 [file formative_v7i1e47325_app2.docx]

**Table S1**. Behavior pattern matching accuracy between recognition number of times by SFM sensor and assumed behavior number of times in scenarios.

| Scenario | Restroom  (num/scenario num) | Bedroom | Other actions |
| --- | --- | --- | --- |
| Scenario 1 | 20/20 | 8/8 | 24/24 |
| Scenario 2 | 24/24 | 20/20 | 44/44 |
| Scenario 3 | 32/32 | 16/16 | 48/48 |
